# Apport de la tomodensitométrie dans le diagnostic de la tuberculose vertébrale à propos de 496 cas à Abidjan

**DOI:** 10.11604/pamj.2015.20.201.5996

**Published:** 2015-03-05

**Authors:** Mariam Gbané-Koné, Baly Ouattara, Mohamed Diomandé, Viva Sessou, Fulgence Kaboré, Kouadio Flore Djoko, Moriba Traoré, Edmond Eti, N'zué Marcel Kouakou

**Affiliations:** 1 Service de Rhumatologie, CHU de Cocody, Abidjan, Côte d'Ivoire

**Keywords:** Mal de Pott, TDM, spondylodiscite, tuberculose, Pott disease, CT scan, spondylitis, tuberculosis

## Abstract

**Introduction:**

Le mal de Pott est la forme la plus fréquente des tuberculoses ostéo articulaires, le diagnostic de certitude reste difficile. L'imagerie tient une place indéniable dans le diagnostic. L'objectif de cette étude était de déterminer la prévalence de la tuberculose vertébrale et l'apport de la TDM dans le diagnostic.

**Méthodes:**

Nous avons mené une étude rétrospective sur dossiers de tuberculose vertébrale dans le service de Rhumatologie du CHU de Cocody de Janvier 2006 à Décembre 2013. N'ont pas été inclus dans l’étude, tous les dossiers ne comportant pas d'imagerie.

**Resultats:**

La prévalence hospitalière de la tuberculose vertébrale était de 10,95%, elle représentait 90,2% de la tuberculose ostéoarticulaire. Elle concernait les 2 sexes, l’âge moyen était de 43,27 ans (4-88ans). L'atteinte dorsolombaire était la localisation la plus fréquente (95,13%). L'atteinte du rachis cervical était rare. La spondylodiscite était fréquente (92,14%). La spondylite (6%) et l'atteinte de l'arc postérieur (0,86%) étaient rares. La spondylodiscite était unique le plus souvent (70,05%), les formes multiétagées ont été notées dans 28,65% des cas, les atteintes mutifocales ont été notées dans14, 63% des cas. Les localisations inhabituelles étaient: atteinte sous occipitale (n=3), atteinte concomitante des 3 segments rachidiens (n=3), atteinte du sacrum (n=1), abcès isolés du psoas (n=4). La prévalence des abcès était de 85,91%, celle des épidurites était de 80,17%. La ponction scannoguidée des abcès a été réalisée dans 20 cas, la recherche de BAAR était positive dans 15 cas. Il existait une tuberculose viscérale évolutive dans 20% des cas.

**Conclusion:**

La TDM est indéniable dans le diagnostic positif et lésionnel de la tuberculose vertébrale. Le retard au diagnostic explique l’étendue des lésions.

## Introduction

L'atteinte vertébrale est la première localisation de la tuberculose ostéoarticulaire (TOA), elle représente 50% des cas [1, 2]. Elle connait actuellement une recrudescence aussi bien dans les pays développés que dans nos pays à forte endémicité tuberculeuse [1–3], où elle demeure un problème de santé publique. Le diagnostic de certitude n'est pas souvent aisé. La TDM contribue largement au diagnostic positif, au bilan lésionnel et à la recherche de complications de la spondylodiscite tuberculeuse [3]. Notre étude a été entreprise afin de déterminer la prévalence et les aspects radiologiques (TDM) du mal de Pott en milieu hospitalier ivoirien.

## Méthodes

Il s'agissait d'une étude rétrospective sur dossiers de patients hospitalisés pour tuberculose vertébrale (TV) de Janvier 2006 à Décembre 2013. Elle a été menée dans le service de Rhumatologie du CHU de Cocody. Le diagnostic de TV a été basé à la fois sur des preuves bactériologiques et ou histologiques mais aussi (le plus souvent), sur un faisceau d'arguments épidémiocliniques, radiologiques et évolutifs (une évolution satisfaisante sous traitement antituberculeux). Les patients n'ayant pas d'imagerie (radiographie standard, TDM et ou IRM) n'ont pas été inclus dans l’étude. Les paramètres sociodémographiques, cliniques et radiologiques ont été étudiés.

## Résultats

### Données épidémiologiques

#### Prévalence

Quatre cent quatre vingt seize dossiers de TV ont été colligés sur un ensemble de 4531 patients hospitalisés dans la même période, soit une prévalence hospitalière de 10,95%. La TV représentait 90,2% de la TOA (550 cas).

#### Age

L’âge moyen de nos patients était de 43,27 ans ± 16,002 (4-88 ans).

### Sexe

Les hommes (53,63%) étaient plus atteints que les femmes (46,37%). Le Sex ratio (H/F) était de 1,16.

### Données cliniques

Un antécédent de tuberculose viscérale a été retrouvé dans 04,6% des cas et une notion de contage tuberculeux dans 26,2% des cas. Le délai d'hospitalisation était supérieur à 3 mois dans 72,14% des cas. La douleur rachidienne a été notée chez tous les patients. Les signes généraux étaient les suivants: l'amaigrissement (81,30%), la fièvre(76,70%), l'asthénie(52,20%), les sueurs nocturnes (44,70%). On notait un effacement de la lordose lombaire et une gibbosité respectivement dans 19,5 et 19,3% des cas. Un syndrome rachidien a été objectivé dans 96,8% des cas et un syndrome radiculaire dans 46,2%. Les complications neurologiques représentaient 16,4% des cas (compression médullaire =11, 3% des cas, compression radiculaire = 04% des cas, syndrome de queue de cheval = 01,1%).

### Imagerie

Le taux de réalisation des différentes imageries était respectivement de 89,8% pour la radiographie standard, de 83,63% pour la TDM (n=460), et de 06,63% pour l'IRM. A la radiographie standard, la spondylodiscite a été suspectée dans 61,54% des cas, la radiographie paraissait normale ou dégénérative dans 38,46% des cas.

### Caractéristiques des lésions à la TDM

#### Niveau de l'atteinte

**Figure 1:**
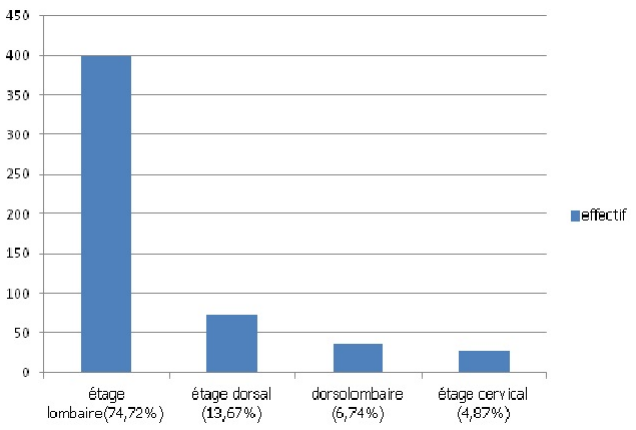
Répartition selon la localisation rachidienne de la tuberculose

Les différentes localisations ont été résumées à la Figure 1. L'atteinte tuberculeuse a intéressé l’étage dorsolombaire dans 95,13% des cas. Les atteintes inhabituelles étaient les suivantes: une atteinte sous occipitale chez 3 patients, une atteinte concomitante des 3 segments rachidiens dans 3 cas et une atteinte du sacrum a été objectivée dans un cas.

#### Multifocalité de l'atteinte

L'atteinte était localisée à un seul étage dans 70,05% des cas, elle était bi-étagée dans 19,51% et elle concernait 3 étages et plus dans 09,14% des cas. L'atteinte était multifocale dans 14,63% des cas.

*Type de l'atteinte* Les principales lésions anatomoradiologiques étaient: La spondylodiscite: (92,14%), des cas La spondylite: (6%), des cas L'atteinte de l'arc postérieur: (0,86%) des cas L'abcès isolé du psoas dans: 4 cas

#### Atteinte des parties molles

**Figure 2:**
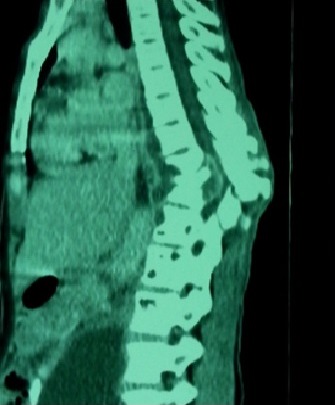
TDM dorsolombaire montrant une image de spondylodiscite à la charnière dorsolombaire avec une épidurite compressive et une déformation en cyphose

**Figure 3:**
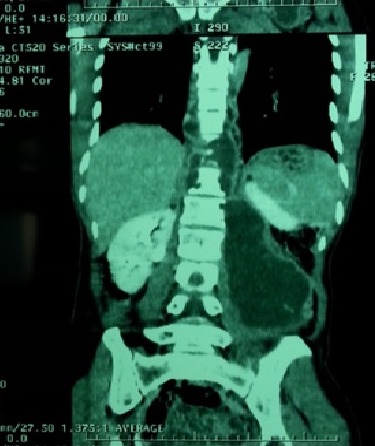
Volumineux abcès du muscle psoas gauche au scanner

Les épidurites ont été notées dans 80,17% des cas et les abcès des parties molles paravertébrales dans 85,91% des cas (Figure 2, Figure 3).

#### Topographie des abcès paravertébraux

Muscles péri vertébraux: 49,4% des cas; Muscles psoas: 18,1% des cas; Muscles retropharyngés: 06,6% des cas. Vingt drainages scannoguidés d'abcès volumineux on été réalisés. La bacilloscopie de ces abcès a mis en évidence les BAAR dans 15 cas. On a noté une compression médullaire dans 11,3% des cas.

#### Bilan d'extension

Une tuberculose viscérale évolutive a été retrouvée dans 20,6% des cas.

## Discussion

La prévalence du mal de Pott était de 10,95% et l'atteinte vertébrale représentait 90, 2% de la TOA dans notre étude. Cette prévalence a doublé en moins d'une décennie dans le service, en effet dans l’étude d'Eti [4] en 2010 sur la tuberculose vertébrale, elle était de 5,1%. La tuberculose vertébrale demeure toujours une maladie d'actualité dans nos pays en voie de développement où elle continue à poser un problème de santé publique [3]. Les conditions socioéconomiques défavorables (précarité, promiscuité, mauvaise hygiène de vie) et l'infection à VIH [1, 2] sont autant de facteurs favorisant l'infection dans nos pays [2, 3]. Aussi grâce à l'amélioration de nos plateaux techniques en imagerie performante (TDM, IRM), le diagnostic est précocement posé à un stade où les radiographies standard paraissent normales [5]. La plupart de nos patients sont des adultes jeunes, avec un âge moyen de 43,27 ans. Dans les pays endémiques, la tuberculose vertébrale touche le plus souvent les adultes entre 40 et 50 ans [3, 6–9]. Dans les pays développés, la tuberculose vertébrale de l´adulte s´observe surtout chez des sujets âgés [1, 10], mais aussi chez des sujets à risque et, de plus en plus souvent, chez des immigrants en provenance de zone d´endémie tuberculeuse. Les hommes étaient autant touchés que les femmes. Il n´y a pas de prédominance de sexe mais plutôt des variations selon les séries [11]. Le principal facteur favorisant est l´existence d´un antécédent tuberculeux, présent chez 04,6% de nos malades contre 17% dans l’étude de Fedoul [8]. La notion d´un contage tuberculeux a été retrouvée dans 26,20% des cas. Dans la littérature, elle varie entre 5 et 23% des cas [11]. La présentation clinique est peu spécifique, l’évolution est insidieuse. Et les signes généraux ne sont pas bruyants, tout cela est responsable d'un délai diagnostic généralement long de 1 à 26 mois [2, 7]. Dans notre série, la douleur rachidienne était le symptôme majeur dans 100% des cas et la majorité des patients consultait après 3 mois.

Le retard diagnostic fait l'unanimité des auteurs [2, 3, 6, 7], expliquant ainsi la fréquence des déficits neurologiques qui sont retrouvés dans des proportions de 20 à 40% [12]. Nos patients consultaient au stade de complications neurologiques dans 16, 4% des cas, la prévalence des complications neurologiques était de 27,2% chez Eti [4]. Le bilan radiologique est capital pour le diagnostic et le bilan d'extension dans le mal de Pott. La radiographie standard est généralement prise en défaut durant les premières semaines de l'infection disco-vertébrale, car ne pouvant détecter une perte de la charge calcique que lorsqu'elle dépasse 50% [5]. Le taux de réalisation de la TDM a doublé en une décennie dans le service. Dans l’étude de Ouattara [9] réalisée en 2004 sur le mal de Pott, ce taux était de 45,16%. Cette hausse s'explique par une meilleure accessibilité à la TDM, en effet l'hôpital s'est doté d'un appareil scanner, et le cout de l'examen a aussi baissé de moitié. La principale localisation rachidienne de la tuberculose, était l’étage dorsolombaire (95% des atteintes vertébrales, avec une prédominance de l'atteinte lombaire). Ce constat est unanimement retrouvé chez tous les auteurs, aussi bien chez les adultes [2, 3, 8, 9, 13] que chez les enfants en effet Mabiala Babela [14] dans une étude au Congo sur le mal de Pott chez les enfants, avait aussi trouvé une prédominance dorsolombaire. Pour Pertuiset [1], les rachis dorsal et lombaire représentent à eux deux, 80% des localisations rachidiennes; et sont à peu près de part égale. L'atteinte cervicale est rare, environ 5% des maux de Pott [3, 9, 13–15]. Cette localisation se singularise par l'atteinte du rachis cervical haut (C1 - C2 - C3) appelé mal de Pott sous-occipital L'atteinte sous occipitale est estimée à moins de 1% de l'ensemble des tuberculoses vertébrales [16]. Elle expose à de graves complications bulbo-médullaires, ce qui nécessite un diagnostic et un traitement rapide. Dans notre série, nous avons eu 3 cas, l'un des patients a eu une présentation particulière à type de syndrome de Brown Séquard.

Habituellement, le mal de Pott peut toucher 2 étages: soit cervico-lombaire, dorso-lombaire ou cervico-dorsal [17]. L'atteinte concomitante des 3 segments rachidiens est rare, seulement 3 cas dans la littérature [18]. Cette présentation exceptionnelle a été notée chez trois patients dans notre étude. Deux patients avaient une localisation viscérale associée (pulmonaire et ganglionnaire). L’évolution a été satisfaisante uniquement sous traitement médical et corset d'immobilisation chez tous les 3 patients. La tuberculose vertébrale chez la plupart de nos patients, a été caractérisée par une localisation unique (70,05%). Ousehal [19] au Maroc, retrouvait également une localisation unique dans 80,3% des cas. Le plus souvent, le mal de Pott intéresse deux vertèbres ou plus de façon contigüe [20]. Des formes multiétagées ont été décrites [2, 14], elles représentaient 09,14% des cas dans notre étude. Pertuiset (1) trouvait 20% de localisations multifocales. Au plan anatomique, la spondylodiscite était la principale lésion. Les lésions tuberculeuses prédominent toujours au niveau de l'arc antérieur des vertèbres, l'atteinte de l'arc postérieur est rare puisque observée. Seulement dans 10 à 16,4% des cas [3, 14, 21]. Les lésions de l'arc postérieur sont mieux vues à la TDM(21), et sont essentiellement ostéolytiques, elles peuvent intéresser tous les éléments de l'arc postérieur (pédicules, lames, apophyses épineuses, apophyses transverses). Les spondylites sont rares [2, 3]. La prévalence des abcès des parties molles était élevée dans l’étude. L´abcès paravertébral est fréquemment rencontré dans le mal de Pott. Sa fréquence est estimée entre 57 à 70% des cas [7]. La présence des abcès surtout volumineux avec des calcifications, est d´une grande valeur diagnostique [5, 7]. Dans le bilan d'extension de nos patients, une tuberculose viscérale évolutive a été retrouvée dans 20,6% des cas. Dans la littérature, l'association d'une localisation extravertébrale au cours du mal de Pott se rencontre dans 20 à 30% des cas, aussi leur présence permet d'orienter le diagnostic [2, 11, 21]. Au cours de ces 2 dernières années, la radiologie interventionnelle à ses débuts a permis de réaliser 20 drainages scannoguidés d'abcès volumineux, dont 15 ont mis en évidence le BK. Il ressort de ces constats que la collaboration entre les services de rhumatologie et de radiologie est nécessaire dans un but diagnostic et aussi pour une meilleure prise en charge des atteintes des parties molles dans laTV.

## Conclusion

L'imagerie constitue incontestablement l'un des piliers du diagnostic du mal de Pott. Cette étude a permis d'illustrer cela, en effet la TDM a permis de préciser les lésions même à un stade précoce de la maladie, de mettre en évidence les abcès et les épidurites mais aussi des localisations inhabituelles (sous occipitales). Elle a permis la réalisation de gestes radioguidés (ponction d'abcès), et a contribué ainsi au diagnostic de certitude de l'infection, enfin la TDM nous a permis de surveiller l’évolution des lésions.
